# Ten Future Challenges in the Field of Transcatheter Mitral Valve Edge-to-Edge Repair

**DOI:** 10.3390/jcm13061799

**Published:** 2024-03-21

**Authors:** Mathias Orban, Ludwig T. Weckbach, Thomas J. Stocker, Philipp M. Doldi, Michael Näbauer, Steffen Massberg, Jörg Hausleiter, Lukas Stolz

**Affiliations:** 1 Medizinische Klinik und Poliklinik I, LMU Klinikum, Marchioninistraße 15, 81377 Munich, Germany; 2 German Center for Cardiovascular Research (DZHK), Partner Site Munich Heart Alliance, 81377 Munich, Germany

**Keywords:** M-TEER, mitral valve edge-to-edge repair, mitral regurgitation, heart failure

## Abstract

Mitral valve transcatheter edge-to-edge repair (M-TEER) and replacement (TMVR) have evolved as guideline-recommended treatment approaches for mitral regurgitation (MR). Even though they are supported by a growing body of evidence from either randomized trials or large registries, there are still several unsolved challenges in the field of interventional MR treatment. In the present review, we discuss the ten most important open questions regarding M-TEER and TMVR.

## 1. Introduction

Within less than 20 years, mitral valve transcatheter edge-to-edge repair (M-TEER) has evolved from an experimental approach, in fact mimicking the surgical Alfieri technique, to a guideline-recommended intervention for relevant mitral regurgitation (MR) [1,2,3]. These recommendations were based on the results of randomized trials, namely the *Endovascular Valve Edge-to-Edge Repair Study* (EVEREST II, which included primary MR patients), the *Cardiovascular Outcomes Assessment of the MitraClip Percutaneous Therapy for Heart Failure Patients with Functional Mitral Regurgitation* (COAPT, which included secondary MR patients), and several large-scale registries [2,4,5,6,7,8,9]. Real-world data have shown similar feasibility, effectiveness, and safety of M-TEER in a non-randomized setting despite differences in study populations [5,9,10,11].

**Figure 1:**
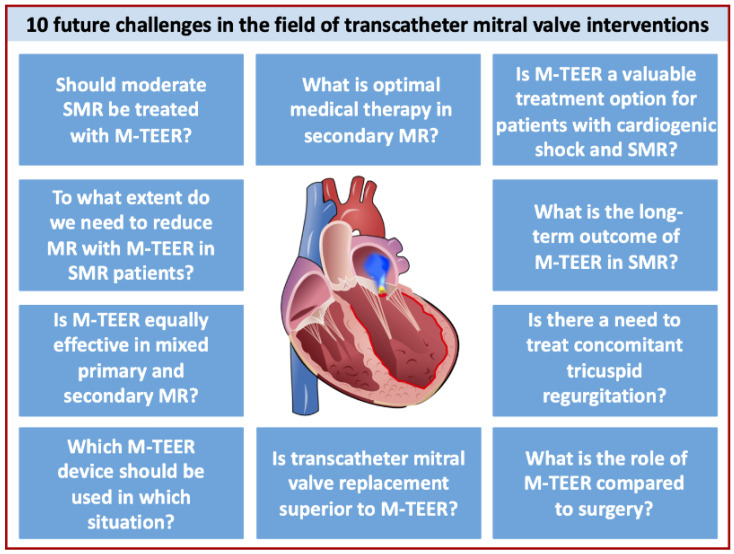
Future challenges in the field of transcatheter mitral valve interventions. We identified 10 major challenges that comprise patient and device selection, procedural aspects and outcome in comparison with surgical options. MR = mitral regurgitation; M-TEER = mitral valve transcatheter edge-to-edge repair; SMR = Secondary mitral regurgitation.

Growing experience has unambiguously demonstrated that patient selection prior to M-TEER is key to optimizing outcomes and, hence, symptomatic and survival benefits [12,13]. Following a number of groundbreaking trials, our pathophysiological understanding of the disease has substantially increased, which has helped improve the identification of the right patient for the right procedure [7]. The latter is of paramount importance in a fast-moving field, with M-TEER being only one solution in the toolbox of repair and replacement devices for both primary MR (PMR) and secondary MR (SMR) [14]. According to the American Heart Association/American College of Cardiology (AHA/ACC) guidelines, M-TEER received a class IIa recommendation in both PMR and SMR patients [15]. Correspondingly, the 2021 European guidelines updated the recommendation level for SMR-TEER to IIa in COAPT-like patients [16]. Nevertheless, there are still clinical challenges for M-TEER, which need to be addressed by ongoing research and future studies. These comprise, i.e., ideal timing of the intervention, optimization and standardization of concomitant medical therapy (and defining guideline-directed medical therapy for SMR), decision-making for simultaneous or sequential non-mitral intervention, improvement in device design for tailoring the intervention, and inter-device comparability for different etiologies. Future research efforts in the field of transcatheter SMR treatment should and can solve those issues. In this review, we aimed to break down these challenges into ten key questions regarding the future of M-TEER (Figure 1).

## 2. 10 Challenges of M-TEER

### 2.1. Should Moderate (Symptomatic) SMR Be Treated with M-TEER?

Data on the natural history of SMR in the setting of sole medical treatment are scarce [17]. These data are key to a better understanding of MR progression from lower grades to more severe grades and might help with identifying the optimal timepoint for interventional and/or surgical treatment. In fact, moderate SMR is associated with impaired quality of life but also reduced survival compared to trivial or mild SMR in conservatively treated patients [18]. Therefore, it could be reasonable to also treat moderate (symptomatic) SMR with mitral interventions to avoid the future progression of SMR and its impact on prognosis. For the surgical repair of SMR in parallel to coronary artery bypass grafting, evidence for a clinical benefit has not been established [19]. In surgically high-risk patients, there might be a potential benefit. This hypothesis is currently tested by the *Transcatheter Mitral Valve Repair for the Treatment of Mitral Valve Regurgitation in Heart Failure* (EVOLVE-MR, NCT03891823) trial, which will assess quality of life over 24 months in patients treated with M-TEER vs. medical therapy for moderate SMR. Although quality of life is a relevant patient-centric endpoint in valvular heart failure trials, it is obvious that the treatment of moderate SMR should not only have a symptomatic but also a beneficial long-term prognostic impact. Therefore, respective trials will require adequate patient numbers and long-term follow-up to uncover the potential effects of M-TEER on survival. In light of this discussion, it is also important to harmonize guideline disparities regarding the definition of different SMR grades across regions [15,20], as this is a major obstacle for comparing trial results and avoiding lengthy discussions about the transferability of these results to different regions.

### 2.2. To What Extent Do We Need to Reduce MR with M-TEER in SMR Patients?

In the randomized COAPT trial, residual mild MR was not associated with improved outcomes when compared to residual mild-to-moderate MR in terms of mortality and hospitalization for heart failure [21]. The same results were reported in a retrospective substudy of the randomized *Percutaneous Repair with the MitraClip Device for Severe Functional/Secondary Mitral Regurgitation* (MITRA-FR) trial, which had a similar setting to the COAPT trial, but showed neutral results in terms of mortality and hospitalization for heart failure in the whole trial population [22]. A possible explanation could be the comparably small sample size of certain subgroups in a randomized-controlled setting, where only half of the patients received a device due to 1:1 randomization. Therefore, subgroup analysis can only be indicative and needs dedicated evaluation in a separate study, or potentially pooling of results. Beyond that, effects might only be observed after longer-term follow-up, especially when differences in clinical benefit are minimal in the short term. In contrast, large SMR-TEER registries (e.g., EURO-SMR or the COAPT-Post Approval Study) have provided evidence that less residual MR is associated with improved outcomes [10,23,24,25]. Further insights into transcatheter mitral valve replacement (TMVR) studies might improve our understanding of how much SMR reduction is needed to improve outcomes, as in these patients, complete elimination of MR is usually obtained [26,27,28]. Maximum reduction in MR often requires implantation of more than one TEER device, which might lead to elevated postprocedural MV inflow gradients. Until today, data on the impact of postprocedural MV gradients in SMR patients have been contradictory, although it seems logical to avoid more than mild mitral stenosis [29,30,31]. As the echocardiographic core lab-controlled substudy of the randomized COAPT trial demonstrated, a relevant number of SMR patients can develop elevated gradients after the procedure, but the clinical outcome is apparently not affected [32]. Therefore, future research should focus on balancing the degree of MR reduction and possible increases in inflow gradients to find a sweet spot to optimize survival and symptomatic improvement.

### 2.3. Is M-TEER Equally Effective in Mixed Primary and Secondary MR?

An MR of mixed etiology (i.e., having characteristics of SMR and PMR) is not well defined. The echocardiographic guidelines state that mixed MR is probably caused by a sequence of events, with a first hit resulting in the primary or secondary MR component and a subsequent second hit leading to the respective other component [33]. This hypothesis has not been validated yet in longitudinal studies; therefore, echocardiographic studies should focus on the natural history of this entity, especially in lower or moderate grades where therapeutic necessity is not yet established. Despite this gap in knowledge, there data that mixed MR patients have different ventricular features, such as less impaired LVEF, than pure SMR patients [10]. M-TEER has in fact been used in mixed MR patients already, but a dedicated analysis of the outcome is missing thus far. A clear distinction between leading pathologies might be difficult, especially in an advanced disease state where atrioventricular structures are already significantly impaired. In the prospective *COAPT-Post Approval Study* (COAPT-PAS), which was intended to investigate real-world results of M-TEER in SMR patients in the United States after the MitraClip device had been approved based on the randomized COAPT results, in fact, up to 39% of patients had features of mixed MR [10]. For those patients, the procedural MR reduction was effective. There are currently no signals that M-TEER could be less effective in mixed MR compared to PMR or SMR. Still, the prognostic effect of mixed MR is unclear. To our knowledge, there is currently no ongoing, dedicated trial for mixed MR patients comparing M-TEER with either GDMT alone or even against mitral valve surgery. As COAPT-PAS has demonstrated, a substantial share of patients undergoing M-TEER have mixed MR, so a stringent analysis of these patients is relevant.

### 2.4. Which M-TEER Device Should Be Used in Which Situation?

In North America and Europe, several M-TEER devices of different sizes have been approved, while technical improvement continues [14]. Although the initial device iterations have been on the market for many years, it is not clear whether patients with particular mitral pathologies would receive an increased benefit from the use of a certain device. The *Edwards PASCAL CLASP IID/IIF Pivotal Clinical Trial* (CLASP IID/IIF, NCT03706833) compares two different M-TEER devices in a non-inferiority setup in more than 1200 patients with either PMR or SMR. The co-primary endpoints include major adverse events, MR reduction, and time to first heart failure hospitalization or death. An in-depth echocardiographic analysis, if conducted, will at least provide some guidance on which anatomies could be more suitable for a certain device design and size. The results from the CLASP IID sub-trial for PMR suggest that both devices (PASCAL and MitraClip) are equally effective, without any differences in clinical endpoints. The results for the SMR substudy of this trial are not published yet but are expected soon. Other real-word registries have not shown conclusive evidence for the benefit of an anatomy-guided device selection, which could be due to the inherent bias of observational studies and retrospective settings [34]. Additional research is also needed to further outline the role of valve replacement in the versatile landscape of MR devices. It is yet unknown which primary strategy, either transcatheter or surgical, is most promising in patients at elevated surgical risk with a complex anatomy and a low probability of MR reduction to ≤1+ [28].

### 2.5. What Is Optimal Medical Therapy in Secondary MR?

Drug therapy for left-sided heart failure patients has evolved over the last few years, with the addition of SGLT-2 inhibitors to betablockers, RAAS inhibitors, and mineralocorticoid receptor antagonists (MRA). Notably, all four drug classes can be administered in parallel, a strategy that has not been investigated in SMR patients in particular. Potentially, GLP1 antagonists, which are now investigated in randomized trials, could become part of heart failure therapy, although a recent paper reports an increase in hospitalizations in HFrEF patients on this therapy [35]. Whether selected drugs or their combination will have a superior effect on SMR that is independent of their effect on heart failure has not been extensively studied. Therefore, the heterogeneity of drug regimens in SMR prevails [36,37,38].

Regarding the path to achieving optimal medical therapy, the results from the randomized COAPT trial and the EURO-SMR registry suggest that a relevant number of patients can up-titrate their heart failure drugs after M-TEER, which might be due to a hemodynamic stabilization of these patients, which can subsequently tolerate higher doses of these beneficial drugs [36,39]. Why up-titration of heart failure drugs is frequently not feasible before M-TEER has not yet been delineated, as specific reasons for these limitations are frequently not published in current registries; however, hypotension and renal dysfunction are believed to be major reasons. Furthermore, intolerance to up-titration of heart failure drugs is difficult to assess in retrospective studies.

In fact, historic data have shown that SMR ameliorates with medical therapy for left-sided heart failure, and although it seems logical that stringent up-titration should be associated with improved outcomes, this has never been shown by a randomized controlled study in a dedicated SMR population. Nevertheless, there is no rationale for arguing against current guideline-directed medical therapy for HFrEF in SMR patients as well.

### 2.6. Is Transcatheter Mitral Valve Replacement as Effective or Superior to M-TEER?

The promise of TMVR is to completely abolish MR, which is rarely achieved with an isolated M-TEER procedure alone. The retrospective data suggest that TMVR is in fact more effective in reducing MR than M-TEER, although clinical outcomes regarding mortality and hospitalization for heart failure have been comparable in a recent non-randomized retrospective study [27]. The evidence to answer this issue may soon be available from the ongoing *Clinical Trial to Evaluate the Safety and Effectiveness of Using the Tendyne Transcatheter Mitral Valve System for the Treatment of Symptomatic Mitral Regurgitation* (SUMMIT, NCT03433274). This randomized prospective trial will assess the effect of either M-TEER or TMVR on survival from heart failure hospitalization as the primary endpoint. Secondary endpoints comprise quality of life and exercise capacity, but in contrast to previous randomized study designs in valvular heart failure, apparently they do not report on death or heart failure hospitalization as separate secondary endpoints.

### 2.7. Is M-TEER a Valuable Treatment Option for Patients with Cardiogenic Shock and SMR?

Cardiogenic shock is one of the deadliest conditions in cardiovascular medicine, with mortality rates up to 50% at 30 days [40]. Notably, cardiogenic shock patients regularly suffer from concomitant relevant MR. Registry data suggest that M-TEER could be associated with better outcomes in patients undergoing M-TEER in cardiogenic shock if compared to conventional therapy in cases of relevant MR [41]. The currently ongoing *Transcatheter Mitral Valve Repair for Inotrope Dependent Cardiogenic Shock* (MINOS, NCT05298124) trial will test this hypothesis, with results expected by 2025. The combined endpoint will include in-hospital all-cause mortality, cardiac transplantation, implantation of durable LVAD, or discharge on palliative inotropic therapy over a 3-month observation period. Especially in these highest-risk patients with cardiogenic shock, new therapeutic approaches are needed to ameliorate the dismal outcome. Registry data showed that M-TEER is a potentially effective bridge-to-transplant strategy in selected patients [42]. A considerable percentage of 22.5% of patients no longer had an indication for heart transplantation because of significant clinical improvement after M-TEER [42].

### 2.8. What Is the Long-Term Outcome of M-TEER in SMR?

Recent updates from the original COAPT trial and other registries have shown 5-year follow-up results on clinical endpoints [2,30]. These results show a substantial mortality and hospitalization rate despite successful MR reduction, which reflects the progressed disease state of enrolled patients. As more than 50% of COAPT trial patients died after 5 years, finding ways to improve long-term outcomes in patients after M-TEER should receive top priority. This also implies optimal identification of patients benefiting prognostically from the procedure. As of now, it is still not well established which SMR patients have a clear survival benefit. The diverging results of the only randomized trials, COAPT and MITRA-FR, have moved the field forwards, especially regarding the profound etiologic workup of SMR patients [8,43]. No hypothesis explaining the prognostic benefit of COAPT versus the lack of it in MITRA-FR has proven to be sufficient on its own. Probably, the diverging results are caused by the selection of a certain subcollective of SMR patients in the COAPT trial with rather disproportionate MR, a lower prevalence of advanced heart failure, and right ventricular dysfunction compared to patients randomized in MITRA-FR [44,45]. With the ongoing *A Clinical Evaluation of the Safety and Effectiveness of the MitraClip System in the Treatment of Clinically Significant Functional Mitral Regurgitation* (Reshape-HF2 NCT05298124) trial, there is currently a third randomized study of M-TEER versus conservative therapy, which could potentially solve the ongoing controversy of which patients have a likely prognostic benefit from M-TEER. Beyond that, a pooled analysis of both the MITRA-FR and COAPT data might shed further light on this apparently contradictory but highly relevant question.

### 2.9. Is There a Need to Treat Concomitant Tricuspid Regurgitation?

Concomitant TR is associated with increased mortality in heart failure patients [46]. In most real-world studies of patients with moderate-to-severe MR, the prevalence of concomitant severe TR reaches up to 20% [10,34]. Tricuspid TEER has been studied now for over a decade and proven to be effective for short- and mid-term TR reduction [47,48]. There is an obvious need to evaluate if additional treatment with TR will improve outcomes in SMR patients. Notably, some studies suggest that impaired RV function could be a more relevant parameter of right-sided heart failure than severe TR, as the latter was not an independent predictor for outcomes in studies that looked at the prognostic role of TR in M-TEER [49,50]. Retrospective 3D echo data from a European single-center study have shown that M-TEER is associated with right ventricular reverse remodeling and a subsequent significant reduction in the severity of concomitant TR [51]. Whether concomitant secondary TR is a bystander or a valid and mandatory treatment target can only be addressed in a randomized controlled trial. To our knowledge, there is no such trial currently recruiting patients.

### 2.10. What Is the Role of M-TEER Compared to Surgery?

For PMR, the first M-TEER device generation could not show benefits over surgery [4]. Whether isolated M-TEER for SMR has advantages over surgery has not been shown by any randomized clinical trial yet. Still, another randomized study, the *Revascularization and Valve Intervention for Ischemic Valve Disease trial* (REVIVE, NCT04822675), will investigate the different outcomes of M-TEER plus coronary artery bypass grafting vs. surgical mitral valve repair or replacement plus coronary artery bypass grafting and thus at least hint at the future role of both methods in patients eligible for both approaches. Due to the high recurrence rates and currently no evidence of survival benefits, the indication for isolated mitral valve surgery in SMR is limited [16]. The results of the MATTERHORN trial, which was intended to randomize patients with severe functional or ischemic MR to either TEER or MV surgery, are awaited soon and might help shed light on this question. The primary study outcomes are a composite endpoint of death, rehospitalization for heart failure, reintervention (repeat operation or repeat intervention), assist device implantation, and stroke (whatever comes first) 12 months post intervention. Beyond the short-term outcomes, it will be extremely important to compare the durability of MR reduction for patients undergoing surgical vs. interventional treatment. Whether M-TEER will supersede isolated mitral valve surgery for SMR as the therapy of choice in real-world clinical practice in the next few years depends on future trials. For PMR patients, several studies are currently investigating the performance of M-TEER vs. surgery (NCT04198870; NCT05051033; NCT03271762).

## 3. Conclusions

The results of several real-word registries and two randomized trials have shown that M-TEER is safe and effective in terms of MR reduction. M-TEER has been incorporated into major guidelines as an important therapy for mitral valvular heart failure [16]. Still, there are many questions to be answered in this research field. These questions concern the selection of patients, optimal procedural approaches, and value against other repair or replacement methods. With the emerging application of artificial intelligence in the field of cardiovascular research, understanding complex disease patterns might be facilitated in the future. More advanced statistical approaches could further level the path towards more individualized risk prediction and treatment selection. As both mature techniques like M-TEER and more recently developed approaches like TMVR need constant improvement, our outlined 10 challenges provide guidance for current and future research in the field of transcatheter mitral valve treatment. Both large multicenter registries as well as well-controlled randomized trials are needed to answer these open questions in a meaningful and valid way.
